# Shaping Future Foods through Fermentation of Side Streams: Microbial, Chemical, and Physical Characteristics of Fermented Blends from Sunflower Seed Press Cake and Cheese Whey

**DOI:** 10.3390/foods12224099

**Published:** 2023-11-12

**Authors:** Norbert Raak, Nicola Mangieri, Roberto Foschino, Milena Corredig

**Affiliations:** 1 Department of Food Science, Aarhus University, 8200 Aarhus N, Denmark; norbert@food.au.dk (N.R.); nicola.mangieri@unimi.it (N.M.); 2 CiFOOD Centre for Innovative Food Research, Aarhus University, 8200 Aarhus N, Denmark; 3 Department of Food, Environmental and Nutritional Sciences, Università degli Studi di Milano, 20133 Milan, Italy; 4 Department of Biomedical, Surgical and Dental Sciences, Università degli Studi di Milano, 20133 Milan, Italy; roberto.foschino@unimi.it

**Keywords:** oilseed, plant protein, milk protein, lactic acid bacteria, yeast, microstructure, storage stability, food waste, by-product, circularity

## Abstract

The current food system suffers from the inefficient use of resources, including the generation of side streams of low economic value that still contain nutritional components. One potential approach to reach a more sustainable food system is to reintroduce such side streams into a circular value chain and valorise them in novel food products, preferably in an unrefined or minimally refined manner. Blending side streams from different industries might be a suitable way to improve the nutritional value of the final matrix. In this study, sunflower seed press cake and cheese whey were combined to obtain matrices containing valuable proteins, structuring polysaccharides, as well as lactose and minerals facilitating fermentation with three different co-cultures of lactic acid bacteria and yeasts. Fermentation for 48 h at 26 °C decreased the pH from ~6.3 to ~4.7 and enhanced the storage stability of the blends with no effect on their rheological properties and microstructure. This research demonstrates the potential of fermentation as a mean to stabilise side stream blends while only minimally affecting their physical appearance.

## 1. Introduction

The development of novel foods and food ingredients from plant-based resources is receiving increasing attention in scientific and industrial research, driven mainly by the intention to reach a more sustainable food system through offering more environmentally friendly alternatives to animal-based products such as meat and dairy. However, many current applications are based on using purified fractions such as protein isolates, where the resource- and energy-intense purification processes add up to the environmental impact of the final product and thereby diminish to some extent the sustainability efforts of using plant-based resources [1]. Moreover, the applied ingredients usually originate from fresh crops, whereas food processing side streams or co-products are still widely overlooked as sources for, e.g., protein ingredients, hydrocolloids, or even native oil bodies [2]. Such side streams are generated when only a part of the raw material is extracted as the main product, leaving residues that are often of lower economic value (e.g., juice production, cheese making, oil pressing, beer brewing). Reintroducing such side streams into the food chain would increase resource efficiency and contribute to reaching a more sustainable, zero-waste food system.

The vision of our research is to develop innovative strategies for incorporating the solid pressing residues from vegetable oil production—so-called “press cakes”—into the food chain, without or with only very limited purification [3]. These materials contain valuable proteins and fibres, and sometimes residual oil in its native form—the so-called oil bodies or oleosomes. Such a holistic approach requires, therefore, a detailed understanding of the function of different bio(macro)molecule constituents as well as the effect of the processing history of the ingredient with regard to structure-forming properties in complex matrices during processing and their techno-functional performance in foods [4,5]. Previous studies showed that lowering the residual oil content increases the water-binding capacity and water swelling of press cakes from sunflower and pumpkin seeds [6]. On the other hand, native oil bodies did not impair the performance of sunflower seed press cake as an emulsifier when compared to a protein-rich fraction, whereas a fibre-rich fraction derived from the press cake showed inferior emulsifying properties in terms of emulsion stability, droplet size, and interfacial film formation [7]. High-temperature treatments (≥120 °C) of sunflower seed press cake dispersions under continuous stirring were shown to result in the formation of protein microparticles dispersed in an aqueous, fibre-rich matrix, whereas a homogeneous network was formed during heating without shear [8].

Many plant-based foods show a lower nutritional quality compared their animal-derived counterparts, which may stem from the lower biological value and/or digestibility of the proteins and perhaps deficiencies of certain micronutrients such as calcium or vitamin B12 [9,10,11]. In this regard, hybrid products containing both plant- and animal-based resources can provide a solution to ensure nutritious and healthy foods [12]. In this research, the oilseed press cakes were blended with cheese whey [3], a side stream from the dairy industry that contains high-quality proteins, calcium, and also lactose [13], which is a suitable carbon source for fermentation. This would also allow for the better fermentation of oilseed press cakes with microorganisms from traditional Western fermented foods (e.g., lactic acid bacteria, yeasts) rather than fungi from traditional Asian cuisines, which might not meet the sensory preferences of European consumers. Even though cheese whey is being valorised in many different ways in large dairy factories, by obtaining lactose, whey protein concentrates and isolates, or by producing bioethanol [14], it is often still a waste stream in small, artisanal cheese factories due to the lack of infrastructure, leaving a potential for utilising cheese whey in novel applications. Our choice to use sunflower seed press cake and cheese whey was furthermore based on the fact that both are considered to be among the major food processing side streams in the European Union (https://eu-refresh.org/top-20-food-waste-streams.html; accessed on 6 November 2023).

Fermentation has been used over centuries to preserve perishable foods such a milk, meat, and vegetables, and has become a well-designed biotechnological process for manufacturing functional foods. Consumers consider fermented foods healthy and natural, favouring new interest in fermentation processes for food manufacture [15]. Recently, it was shown that the fermentation of pea protein concentrates by lactic acid bacteria prior to high-moisture extrusion can reduce pea off-flavours, generate umami flavours, and improve the texture of plant-based sausages [16]. Furthermore, the content of the antinutritional compound chlorogenic acid in sunflower protein concentrate was decreased by fermentation with *Lactobacillus helveticus* [17]. This further demonstrates the great potential of fermentation for the manufacture of plant-based food products with acceptable sensory properties. However, it was also suggested to apply assemblies of multiple microbial cultures rather than individual strains to obtain the best results in terms of flavour development [18]. In previous studies, conditions for the controlled [19] and spontaneous fermentation [20] of blends of sunflower seed press cakes and cheese whey were screened, and it was concluded that co-cultures of different lactic acid bacteria (*Lactococcus lactis* or *Lacticaseibacillus paracasei*) and yeast strains (*Kluyveromyces lactis* or *Kluyveromyces marxianus*) are most promising to ferment this substrate under controlled conditions.

The aims of this study were to (1) monitor in detail the fermentation kinetics of blends of sunflower seed press cake and sweet cheese whey by co-cultures of lactic acid bacteria and yeasts in terms of the acidification, cell growth, and metabolisation of sugars; and to (2) investigate if the fermentation affects the structure and physical properties of the blends. The samples were analysed immediately as well as after 7 d of cold storage to verify their microbial and physical stability.

## 2. Materials and Methods

### 2.1. Sample Preparation

Fermentation trials were carried out on cold pressed cake from dehulled sunflower seeds (Schalk Mühle GmbH & Co KG, Ilz, Austria), which was dispersed at a concentration of 225 g/kg in reconstituted whey (60 g/kg whey powder in demineralised water; Bayerische Milchindustrie eG, Palting, Germany) using a kitchen blender (mixer HR1570; Philips Electronics N.V., Amsterdam, The Netherlands). The exact composition of the powders was reported elsewhere [8]. The blend (260 g/kg dry matter; 110 g/kg protein; 95:5 sunflower protein:whey protein ratio) was heated for 5 min at 121 °C in an autoclave (SS-325; TOMY Kogy Co., Ltd., Tokyo, Japan) and cooled at ambient temperature (~21 °C), followed by splitting aliquots of 30 g into sterile centrifugation tubes.

Three different co-cultures (B12L2, B12L7, and B8L2) were inoculated, each formed by one lactic acid bacteria strain (*Lactococcus lactis* strain B12 or *Lacticaseibacillus paracasei* strain B8) and one yeast strain (*Kluyveromyces lactis* strain L2 or *Kluyveromyces marxianus* strain L7). These strains were previously isolated, characterised, and deemed suitable for fermenting the target substrate [19]. Suspensions of fresh cells of each bacterial strain at approx. 10^9^ CFU/mL were obtained via cultivation in BD Difco^TM^ Lactobacilli MRS broth (Thermo Fisher Scientific, Waltham, MA, USA) at 30 °C for 48 h in static conditions, whereas suspensions of fresh yeast cells at approx. 10^8^ CFU/mL were obtained by cultivation in yeast peptone dextrose broth (Merck KGaA, Darmstadt, Germany) at 26 °C for 48 h under agitation (120 rpm). The cells in the suspensions were enumerated by the plate count method as described in Section 2.2.2. Aliquots of fresh cultures were added to the samples at time zero to attain concentrations of 1 × 10^6^ CFU/g lactic acid bacteria and 1 × 10^5^ yeasts and mixed using the kitchen blender (mixer HR1570; Philips Electronics N.V.) after sterilising the mixing tools in an autoclave (SS-325; TOMY Kogy Co., Ltd.). Fermentation was performed for 48 h in a heating chamber (Memmert GmbH+Co.KG, Schwabach, Germany) at 26 °C in static conditions. Sample aliquots were withdrawn after 12, 24, and 48 h for further analyses carried out immediately and after storage at 4 °C for 7 d. Samples referred to as “0 h” were unfermented blends without the co-cultures added unless stated otherwise.

### 2.2. Microbial Analyses

#### 2.2.1. pH Development

The pH during fermentation was recorded using a pH meter with memory function (InoLab 7310 pH meter; WTW GmbH, Weilheim, Germany) equipped with a SenTix 82 electrode (WTW GmbH). The electrode was immersed in the sample inside a centrifugation tube, which was sealed with a plastic film and placed in a heating chamber at 26 °C for 48 h. The measurement interval was set to 15 min. The sample used for monitoring the pH development was not used for further analyses.

#### 2.2.2. Cell Counts

The cell counts of lactic acid bacteria, yeasts, mould contaminants, and mesophilic aerobic bacterial contaminants were determined using the plate count technique. Lactic acid bacteria were counted on BD Difco^TM^ MRS agar (Thermo Fisher Scientific) after incubation at 30 °C for 48 h, yeasts and mould contaminants were cultivated on Yeast Extract Glucose Chloramphenicol (YGC) agar (Merck KgA, Darmstadt, Germany) at 26 °C for 72 h, and bacterial contaminants were enumerated on sugar-free agar medium after incubation at 30 °C for 48 h. The results are presented as the decimal logarithm of the colony-forming units per g of samples (Log_10_ CFU/g).

### 2.3. Chemical Analyses

#### 2.3.1. Degree of Protein Hydrolysis

The degree of protein hydrolysis was assessed as the free amino groups determined using the *o*-phthaldialdehyde (OPA) spectrophotometric assay. First, 10 mg of freeze-dried sample was mixed with 1 mL of PBS buffer (50 mmol/L NaH_2_PO_4_, 100 mmol/L NaCl, pH 7.4), and 50 µL of the mixture was blended with 80 µL of 5% trichloroacetic acid. The samples were kept in ice water for 30 min and subsequently centrifuged at 10,000× *g* and 4 °C for 20 min. Then, 10 µL of each supernatant was transferred to a well in a micro-titre plate and mixed with 200 µL of OPA reagent (5.96 mmol/L OPA, 0.1 mol/L Na_2_B_4_O_7_, 1 g/L sodium dodecyl sulphate, 5.7 mmol/L dithioerythritol). After 15 min at ambient temperature (~21 °C), the absorbance was measured at 340 nm. PBS buffer was used as a blank and treated in the same way as the samples.

A calibration curve with L-leucine in the concentrations of 0–10 mmol was used to calculate the amount of free amino groups as mmol L-leucine equivalents per mg of freeze-dried sample from the measured absorbance.

#### 2.3.2. Gel Electrophoresis

The fermented side stream blends were analysed for their cross-linked as well as hydrolysed protein fractions using denaturing and reducing gel electrophoresis as described previously [8]. Briefly, the samples were diluted 1:50 (*w*/*v*) in demineralised water, homogenised using a vortexter (IKA Werke GmbH & Co.KG, Staufen, Germany), mixed 6.5:2.5:1 (*v*/*v*) with NuPAGE^TM^ LDS buffer and NuPAGE^TM^ reducing agent, and subsequently heated at 95 °C for 5 min in a thermoshaker (IKA Werke GmbH & Co.KG). Aliquots of 7 µL were injected to a NuPAGE^TM^ precast gradient gel (4 to 12% polyacrylamide) placed in an XCell SureLock^TM^ Mini-Cell filled with NuPAGE^TM^ MES SDS running buffer. The separation was run at 200 V for 35 min before staining the gels in SimplyBlue^TM^ SafeStain solution for 2 h and rinsing them in demineralised water for 24 h to remove excess dye. All supplies were obtained from ThermoFisher Scientific (Waltham, MA, USA). A molar mass standard (Precision Plus Protein^TM^, Bio Rad Laboratories, Inc., Hercules, CA, USA) was used for band identification, and the gels were scanned using a ChemiDoc XRS+ gel imaging system (Bio Rad Laboratories, Inc.).

#### 2.3.3. Determination of Sugars

The contents of sugars in the samples before and after fermentation were quantified using high performance anion exchange chromatography–pulsed amperometric detection (HPAEC–PAD) according to a method described by Viola et al. [21] with slight modifications. The samples were freeze-dried and diluted 1:20 (*w*/*v*) with 80% ethanol, followed by heating at 80 °C for 1 h with periodic mixing using a vortexer. After centrifugation (16,000× *g*, 10 min, 1 °C), the supernatants were removed, and the procedure was repeated on the pellet. All supernatants were vacuum dried at 50 °C in a SP Genevac EZ-2 plus and subsequently resuspended in MilliQ water. The samples were centrifuged again, and the supernatants were used for analysis after appropriate dilution.

The analyses were performed using a Dionex ICS-6000 (ThermoFisher Scientific, Waltham, MA, USA) equipped with a CarboPack PA100 IC column (ThermoFisher Scientific). First, 200 mmol/L NaOH was used as eluent and run at an isocratic flow rate of 0.25 mL/min. The injection volume was 10 µL, and the measurements were run at 25 °C. AgCl was used as the reference electrode for analyte detection. Chromeleon v7.3 (ThermoFisher Scientific) was used to record and analyse the data. Lactose, sucrose, raffinose, and galactose concentrations were calculated based on the peak areas and using calibration curves ranging from 0.01 to 0.1 mg/mL.

#### 2.3.4. Total Acidity

The total acidity of the side stream blends was determined through the potentiometric titration of a 10 g side stream blend with 0.25 mol/L NaOH. The results are given as mL of NaOH necessary to reach pH 8.3. The pH was adjusted using an InoLab 7310 pH meter (WTW GmbH).

### 2.4. Physical Analyses

#### 2.4.1. Rheological Properties

A stress-controlled rheometer AR-G2 (TA Instruments, New Castle, DE, USA) equipped with a 40 mm stainless steel, cross-hatched parallel plate geometry, and a Peltier element for temperature control was used to characterise the viscoelastic properties of the fermented and unfermented blends. Measurements were carried out at 4 °C, and the gap width was set to 1 mm. The samples were stored refrigerated prior to the analysis and first equilibrated in the geometry for 1 min, followed by a frequency sweep from f = 100 to 0.1 Hz at a strain amplitude of 0.1% and a strain sweep from 0.1–1000% at a frequency of 1 Hz. Data were collected and analysed using Rheology Advantage v5.7.0 (TA Instruments). The slope of the G’ in the log–log frequency sweeps was calculated as the exponent *n* of the power law function G’(f) = k·f^n^. The flow transition index was calculated from the strain sweep data as the ratio of strain at the end of the linear viscoelastic region (G’ decreased by 5%) and the strain at the cross-over of G’ and G’’ [22].

#### 2.4.2. Colour Measurements

The colour of the side stream blends was measured before and after 48 h of fermentation using a CR400 chromameter (Konica Minolta Sensing Europe B.V., Nieuwegein, The Netherlands). Colour parameters are reported in the CIELAB colour space: L* for lightness, a* for green/red, and b* for yellow/blue.

#### 2.4.3. Microstructure

A 2 mg/mL solution of fluorescein isothiocyanate (FITC) dissolved in acetone was mixed with the blends at a ratio of 1:500 (*v*/*w*), and samples were incubated at room temperature and under light-free conditions for at least 30 min to achieve sufficient protein staining. The samples were placed on glass slides and analysed using a confocal laser scanning microscope (Nikon C2, Nikon Instrument Inc., Tokyo, Japan). The wavelength of the laser was 488 nm, and pictures were taken at a magnification of 20×. Representative images are shown.

### 2.5. Statistical Analysis

All samples were prepared in duplicate. Two-way analysis of variance (ANOVA) was used to analyse statistically significant effects of the co-culture and fermentation time, and a Student’s *t*-test was used to identify statistically significant differences after 7 d of storage at 4 °C. The statistical acceptance level was *p* < 0.05.

## 3. Results and Discussion

### 3.1. Fermentation Kinetics

The side stream blends were fermented with three different co-cultures, where *Lactococcus lactis* strain B12 and *Kluyveromyces lactis* strain L2 were each used in two trials, and *Lacticaseibacillus paracasei* strain B8 and *Kluyveromyces marxianus* strain L7 were each used in one trial. These co-cultures, referred to as B12L2, B12L7, and B8L2 to indicate their composition, were selected based on previous work [19].

#### 3.1.1. Cell Growth of Lactic Acid Bacteria and Yeasts

**Figure 1:**
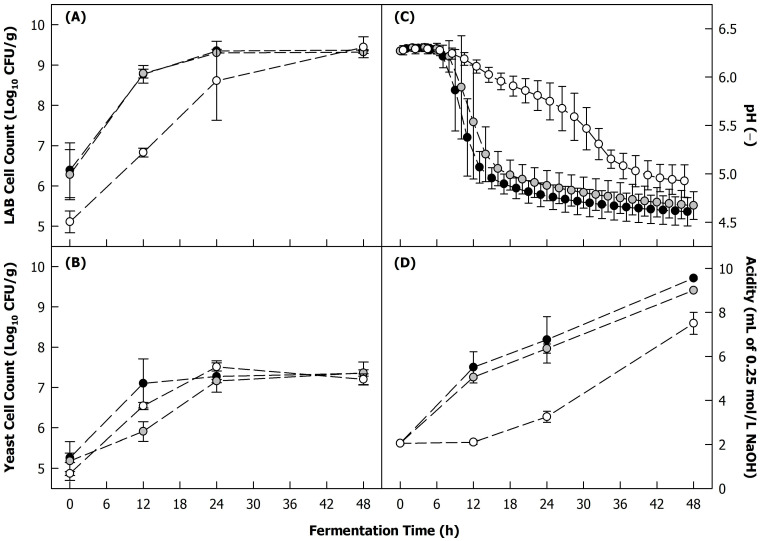
(**A**) Cell count of lactic acid bacteria (LAB) and (**B**) yeasts, (**C**) pH, and (**D**) total acidity of heat-treated (121 °C, 5 min) 225 g/kg sunflower seed press cake in reconstituted sweet whey (60 g/kg) during fermentation with different co-cultures of LAB (*Lactococcus lactis* strain B12; *Lacticaseibacillus paracasei* strain B8) and yeasts (*Kluyveromyces lactis* strain L2; *Kluyveromyces marxianus* strain L7) at 26 °C: B12L2 (black), B12L7 (grey), B8L2 (white). Note: the samples at 0 h were with the co-cultures added.

The growth kinetics of lactic acid bacteria and yeasts during the fermentation of the blends are shown in Figure 1A and Figure 1B, respectively. The cell counts of *Lactococcus lactis* strain B12 increased significantly from ~6.3 to ~8.8 Log_10_ CFU/g during the first 12 h of fermentation, whereas their change with further incubation up to 48 h was not significant. The cell counts of *Lacticaseibacillus paracasei* strain B8 at the starting time were lower than expected based on the inoculation concentration, indicating that some components in the blend, probably phenolic compounds, might have acted negatively for this strain [23]. Consequently, the increase in the cell count of *Lacticaseibacillus paracasei* strain B8 was delayed compared to that of *Lactococcus lactis* strain B12, but was no longer significantly different at 24 and 48 h. Both *Kluyveromyces lactis* strain L2 and *Kluyveromyces marxianus* strain L7 had comparable growth kinetics, although the latter had a significantly lower cell count after 12 h of fermentation, suggesting slightly slower growth. At the end of the fermentation, the cell count of the yeasts was increased from ~5.0 to ~7.3 Log_10_ CFU/g in all trials. It is worth noting that there was no significant effect of the co-culture; both *Lactococcus lactis* strain B12 and *Kluyveromyces lactis* strain L2 showed very similar growth kinetics regardless of their microbial companion, indicating that there was no synergistic or antagonistic effect on the cell growth coming from co-fermentation with another microorganism.

#### 3.1.2. Acidification Kinetics

The pH development during fermentation is illustrated in Figure 1C and generally followed the trend of the cell growth of lactic acid bacteria (Figure 1A) in an inverse manner. After a lag time of about 6 h for all co-cultures, where cells adapted themselves to the new environmental conditions and the pH remained constant at ~6.3, the pH started to decrease due to the metabolic release of organic acids (especially lactic acid). The pH decrease was significantly delayed in the case of fermentation with B8L2, likely due to the slower growth of *Lacticaseibacillus paracasei* strain B8. In contrast, a rapid decrease to pH ~5.3 was observed within the first 12 h of fermentation with B12L2 and B12L7. After 48 h, the pH was decreased to ~4.7 in all trials with no significant differences between the different co-cultures investigated. The total acidity was negatively correlated to the pH (Spearman correlation coefficient = −0.928, *p* < 0.05). It is worth noting though that, unlike for the pH, the total acidity of the blend fermented with B8L2 after 48 h was significantly lower than those of the blends fermented with B12L2 and B12L7, demonstrating that a smaller amount of acidity was formed during this trial, which was not reflected in the pH due to the buffering capacity of the substrate. The effect might also be related to the heterofermentative nature of *Lacticaseibacillus paracasei* strain B8 compared to the obligate homofermentative metabolism *Lactococcus lactis* strain B12. This means that *Lacticaseibacillus paracasei* strain B8 was also able to use pentoses to produce lactate, acetic acid, or ethanol through the pentose phosphate pathway [24], whereas the *Lactococcus lactis* strain B12 could only metabolise hexoses to lactic acid via the Embden–Meyerhof–Parnas pathway. As lactic acid (p*K*_a_ = 3.85) is a stronger acid than acetic acid (p*K*_a_ = 4.75), a higher ratio of acetic acid to lactic acid would result in lower acidity.

#### 3.1.3. Growth of Microbial Contaminants

In the freshly fermented blends at 12, 24, and 48 h, the mesophilic aerobic bacterial contaminants were below the detection limit (<2 Log_10_ CFU/g) for all samples except for that fermented with B8L2 for 48 h, where cell counts of 3.15 ± 1.63 Log_10_ CFU/g were found. These were most likely heat-resistant bacterial spores that were derived from the press cake matrix [20] and that were not inactivated by the adopted thermal treatment. Due to the delayed acidification (Figure 1C), the spores were able to germinate into vegetative cells during the incubation time and could then be detected at the tested dilutions. This underlines the importance of a rapid pH decrease during the fermentation of plant materials to ensure the microbial stability of the resulting product. Mould counts were lower than the detection limit in all samples, as previously reported by Mangieri et al. for microcosms fermented with the same strains in similar conditions [19].

#### 3.1.4. Metabolisation of Sugars

**Figure 2:**
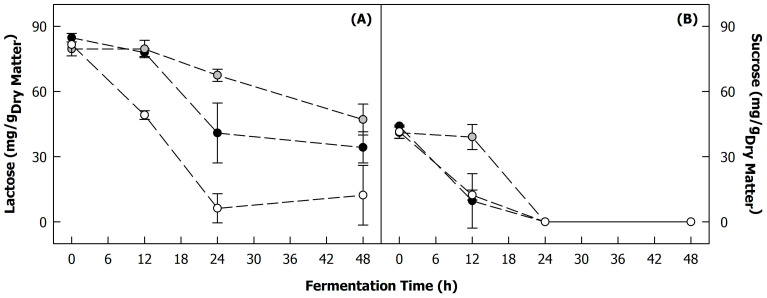
Changes in (**A**) lactose and (**B**) sucrose concentration of heat-treated (121 °C, 5 min) 225 g/kg sunflower seed press cake in reconstituted sweet whey (60 g/kg) during fermentation with different co-cultures of lactic acid bacteria (*Lactococcus lactis* strain B12; *Lacticaseibacillus paracasei* strain B8) and yeasts (*Kluyveromyces lactis* strain L2; *Kluyveromyces marxianus* strain L7) at 26 °C: B12L2 (black), B12L7 (grey), B8L2 (white).

The decreases in the lactose and sucrose concentrations during the fermentation of the blends resulting from the metabolic activities of the co-cultures are shown in Figure 2A and Figure 2B, respectively. The differences between the lactose concentrations in samples fermented with B12L2 and B12L7 were not significant, indicating that both co-cultures consumed lactose at a similar pace. In contrast, B8L2 metabolised lactose significantly faster, although the acidification of these samples occurred much more slowly (Figure 1C,D). This might be because the growth of *Lacticaseibacillus paracasei* strain B8 was considerably challenged (Figure 1A), and lactose might instead have been used by the lactose-positive yeast *Kluyveromyces lactis* strain L2 to produce other metabolites than organic acids. This might suggest a potential competitive interaction for the carbon source between the lactic acid bacteria strain and the yeast strain in this co-culture.

The lactose concentration decreased from ~82 to ~12 mg/g_dry matter_ for samples fermented with the B8L2 and to ~40 mg/g_dry matter_ in the case of fermentation with B12L2 and B12L7 (Figure 2A), demonstrating that the amount of lactose brought into the substrate through the side stream blending was sufficient to facilitate the growth of the co-cultures for reaching an appropriate pH decrease. Furthermore, the sucrose concentration decreased significantly during fermentation (Figure 2B), although there was a lag in the sucrose utilisation in the case of the fermentation with B12L7, which might be related to the slightly lower growth rate of *Kluyveromyces marxianus* strain L7 (Figure 1B). After 24 h of incubation, sucrose was no longer detected in the blends regardless of the co-culture used.

It is worth noting that raffinose and galactose were also found in the unfermented samples at concentrations of ~6 and ~1 mg/g_dry matter_, respectively. However, the concentrations were too low to quantify their decrease during fermentation reliably with the applied method.

### 3.2. Effect of Fermentation on Sunflower Proteins

**Figure 3:**
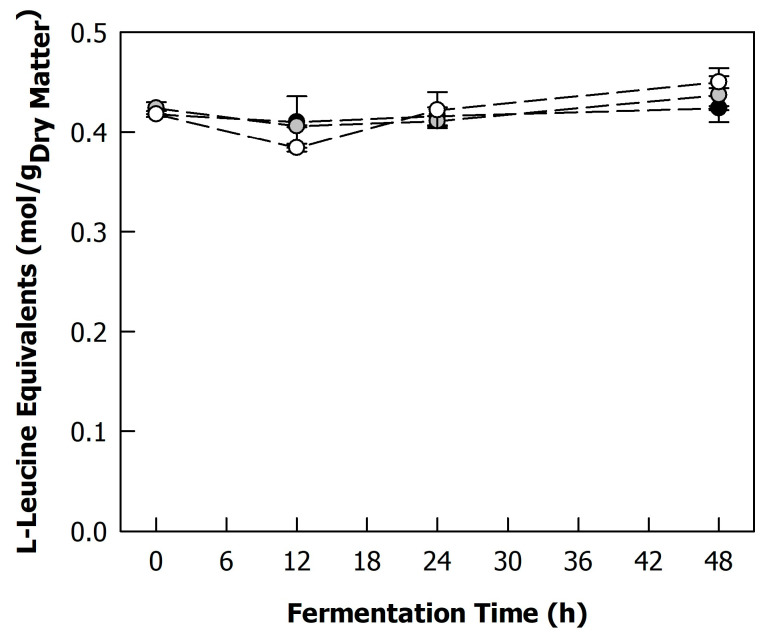
Free amino groups of heat-treated (121 °C, 5 min) 225 g/kg sunflower seed press cake in reconstituted sweet whey (60 g/kg) during fermentation with different co-cultures of lactic acid bacteria (*Lactococcus lactis* strain B12; *Lacticaseibacillus paracasei* strain B8) and yeasts (*Kluyveromyces lactis* strain L2; *Kluyveromyces marxianus* strain L7) at 26 °C: B12L2 (black), B12L7 (grey), B8L2 (white). Free amino groups are expressed of L-leucine equivalents.

To verify if the co-cultures used for fermentation showed any proteolytic activity, the blends were evaluated with regard to free amino groups as well as the protein composition. Figure 3 illustrates the free amino groups in the samples expressed as L-leucine equivalents. The content of free amino groups of the unfermented blend was ~0.42 mol/g_dry matter_. There seems to be a slight trend towards a decrease in free amino groups with fermentation for 12 h, probably due to the early assimilation of available nitrogen by both LAB and yeasts, followed by a slight increase, which could point towards some limited hydrolysis of the proteins. However, the values were not significantly different, with the exception of the blend fermented with B8L2 at 12 and 48 h. Hence, a proteolytic effect of the fermentation could not be fully proven. In a previous work, Pöri et al. detected a strong increase in free amino acids upon the fermentation of sunflower protein concentrate with *Lactobacillus helveticus* [17]. In particular, the content of free glutamic acid was dramatically increased, which was important in terms of flavour development for their application in plant-based meat analogues. *Lactobacillus helveticus* is known to have an efficient proteolytic system [25], which was not the case for the lactic acid bacteria strains used in this study at the tested experimental conditions [26]. Furthermore, meat-like flavours were not desired in the present work, and flavour formation is rather achieved through secondary metabolites of the lactic acid bacteria and yeasts, such as organic acids, alcohols, and aldehydes.

**Figure 4:**
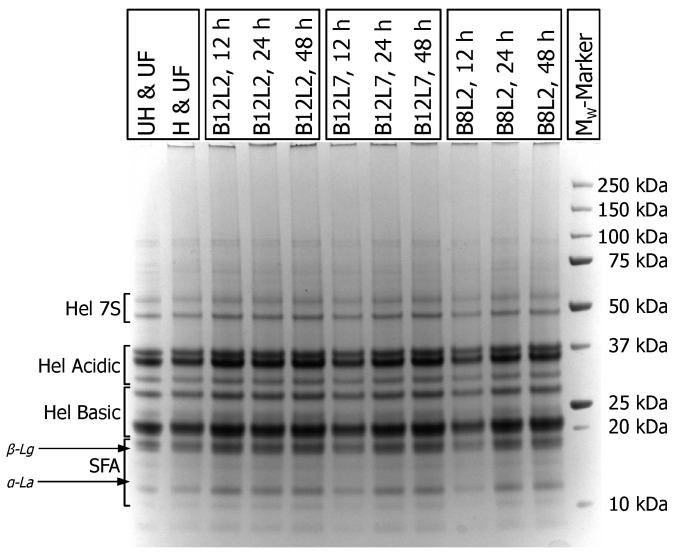
Denaturing and reducing gel electrophoresis of 225 g/kg sunflower seed press cake in reconstituted whey (60 g/kg). Samples were unheated and unfermented (UH and UF); heated (121 °C, 5 min) and unfermented (H and UF), or fermented with different co-cultures of lactic acid bacteria (*Lactococcus lactis* strain B12; *Lacticaseibacillus paracasei* strain B8) and yeasts (*Kluyveromyces lactis* strain L2; *Kluyveromyces marxianus* strain L7) for 12, 24, or 48 h at 26 °C. Different protein fractions are indicated: Hel acidic—helianthinin acidic polypeptides, Hel basic—helianthinin basic polypeptides, SFA—sunflower albumins, Hel 7S—helianthinin trimeric subunits. Expected band positions of α-lactalbumin (α-La) and β-lactoglobulin (β-Lg) are shown based on a previous study [8].

The protein composition of the blends before and at different time points of the fermentation was analysed using gel electrophoresis to evaluate potential proteolysis (Figure 4). The electrophoretic patterns show mainly the sunflower protein fractions, as the whey protein concentration was too low to obtain clearly distinguished bands (~95:5 sunflower protein/whey protein ratio). Previous work on blends of sunflower proteins and whey proteins showed that β-lactoglobulin and α-lactalbumin overlaid with some of the sunflower albumins, whereas bovine serum albumin was found as a distinct band above the trimeric helianthinin fraction [8]. Even though there were some differences in the overall band intensities due to sampling, it can be seen that no major bands disappeared, and no new bands were formed as a result of protein degradation, confirming the conclusions from the evaluation of the free amino groups (Figure 3), pointing to very little proteolysis during fermentation. On the other hand, a band at the top of all lanes, which was absent in the unheated blend, indicated the presence of heat-induced cross-linked protein aggregates [8].

### 3.3. Rheological Properties of Fresh Fermented Blends

Samples withdrawn at different fermentation times were kept in a refrigerator before analysing the rheological properties at 4 °C. As the cold storage slowed down the fermentation considerably, these blends were considered “fresh samples”. The properties of the samples stored for 7 d are discussed later.

**Figure 5:**
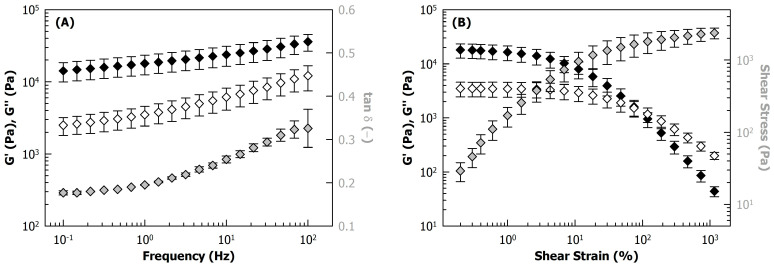
(**A**) Frequency sweep and (**B**) strain sweep experiment of heated (121 °C, 5 min), unfermented 225 g/kg sunflower seed press cake in reconstituted whey (60 g/kg) showing storage modulus (G’; closed symbols) and loss modulus (G’’; open symbols), and, in grey, loss factor (tan δ; **A**) and shear stress (**B**).

The frequency dependency and yield behaviour of the side stream blends are shown in Figure 5A and B, respectively, using the example of the heated, unfermented blend.

#### 3.3.1. Small Deformation Properties

**Figure 6:**
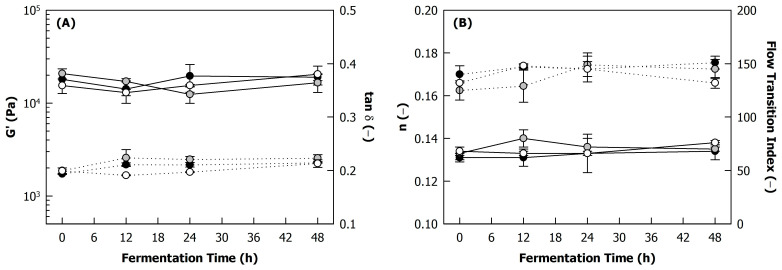
(**A**) Storage modulus (G’; full lines) and loss factor (tan δ; dotted lines) at 1 Hz as well as (**B**) slope of G’ in frequency sweeps (*n*; full lines) and flow transition index (dotted lines) of heat-treated (121 °C, 5 min) 225 g/kg sunflower seed press cake in reconstituted sweet whey (60 g/kg) during fermentation with different co-cultures of lactic acid bacteria (*Lactococcus lactis* strain B12; *Lacticaseibacillus paracasei* strain B8) and yeasts (*Kluyveromyces lactis* strain L2; *Kluyveromyces marxianus* strain L7) at 26 °C: B12L2 (black), B12L7 (grey), B8L2 (white).

In the examined frequency range, G’, G’’, as well as the loss factor tan δ, increased weakly with increasing oscillation frequency (Figure 5A), which is typical behaviour of viscoelastic solids such as gels, pointing to a three-dimensional particulate network structure filled with a liquid [27,28,29]. Figure 6A shows the G’ and tan δ of the side stream blends at 1 Hz as a function of the fermentation time. The G’ of the unfermented blends was approx. 18,000 Pa, which did not change significantly during fermentation. In protein-based systems, acidification, as obtained during fermentation (Figure 1C), can result in gel formation followed by a strengthening of the gel network due to an increase in the number of linkages, as a pH decrease lowers the electrostatic repulsion between the protein particles present in the matrix. In the present study, a heat-induced particle gel was already formed during the autoclaving step prior to the fermentation, which is in agreement with previous reports [8]. This might explain why the acidification during fermentation did not further affect the rheological properties. On the other hand, Campell et al. showed for soy proteins that acidification with glucono-δ-lactone after heat-induced gelation increased G’ due to reduced electrostatic repulsion between the protein aggregates [30]. In fact, numerous reports point to the need for heating as a precursor for the acid-induced gelation of plant proteins [31,32]. However, the side stream blends used in this work were not only composed of proteins as the main building blocks but also contained fibres and small amounts of starch, which undergo colloidal changes during the heat treatment [8] and will thus contribute to structuring the heat-induced gels due to interactions with the proteins. The protein aggregates in this complex network structure might therefore be less free to rearrange as the pH decreases during fermentation.

The frequency dependency of G’ was fitted to a power law relationship G’(f) = k·f^n^, where f is the oscillation frequency, k is a proportionality coefficient corresponding to G’ at f = 1 Hz, and the exponent *n* corresponds to the slope of G’ with frequency in a log–log plot (see Figure 5A). Neither tan δ (Figure 6A) nor *n* (Figure 6B) were significantly affected by the fermentation. Both parameters are closely related, as was shown previously for various viscoelastic solids [29,33], and they provide information on the elasticity of a sample. All samples showed *n* << 0.5 (Figure 6B), meaning that their frequency dependency was low, as is typically the case for elastic networks with covalent and/or strong non-covalent cross-links such as gels or doughs. However, the increase in tan δ with increasing frequency (Figure 5A) indicated that the viscous properties of the solvent phase become dominant over the elasticity of the network at higher oscillation frequencies, where both G’ and G’’ increase with slopes of ~1 [27,34].

#### 3.3.2. Large Deformation Properties

With increasing strain amplitude, G’ and G’’ showed first a short plateau, referred to as the linear viscoelastic region (LVR), followed by a gradual decrease and ultimately a cross-over of both moduli (Figure 5B). This experiment characterises the fracture and yield properties of the samples. G’ and G’’ gradually decreased with increases in the strain amplitude over several decades and still showed values >10 Pa even at 1000% strain. This behaviour indicates that the material is flowing rather than fracturing, which would cause a more sudden decrease in the moduli. This is further demonstrated by the flow transition index, which was calculated as the ratio of the strain at the cross-over of G’ and G’’ and the strain at the end of the LVR (see Figure 5B) [22]. The end of the LVR was thereby defined as the strain amplitude, where G’ decreased to 5% of its initial value. The flow transition index of all samples was around 140, with no significant effect of fermentation time and co-culture (Figure 6B), which was rather high compared to a previous study on pea protein and mung bean protein gels, where values of ~10–20 were reported [35]. This suggests that the plastic deformation of the fermented and unfermented side stream blends occurred over a wide range of strain amplitudes and shows the high resilience of the structures due to the coexistence of two phases, i.e., hydrated protein particles and polysaccharides, which are getting aligned with the shear and start flowing at increased amplitudes.

**Figure 7:**
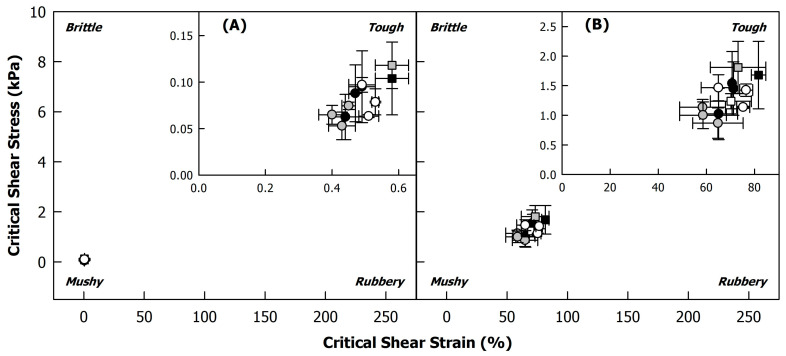
Texture maps of heat-treated (121 °C, 5 min) 225 g/kg sunflower seed press cake in reconstituted sweet whey (60 g/kg) during fermentation with different co-cultures of lactic acid bacteria (*Lactococcus lactis* strain B12; *Lacticaseibacillus paracasei* strain B8) and yeasts (*Kluyveromyces lactis* strain L2; *Kluyveromyces marxianus* strain L7) at 26 °C: B12L2 (black), B12L7 (grey), B8L2 (white). Critical shear stress and critical strain were extracted from strain sweeps at (**A**) the end of the linear viscoelastic region or (**B**) the cross-over of G’ and G’’ (see Figure 5B). Unfermented samples are illustrated as squares; fermented samples at t_12_, t_24_, and t_48_ are illustrated as circles. Insets show magnifications of the bottom left corners.

A frequently used approach to evaluate changes in the large deformation properties of a material, induced, for instance, by processing or variations in composition, is a texture map, where the critical shear stress is plotted against the critical shear strain, and each corner of the plot is attributed to a specific texture attribute (mushy, rubbery, brittle, tough) [36,37,38]. Critical shear stress and strain can thereby be taken at both the end of the LVR and the cross-over of G’ and G’’, which might result in different observations [37]. Figure 7 illustrates both versions of the texture map for the side stream blends in this study. As the assignment of the samples to the texture attributes in each corner is highly dependent on the scaling of the axes, the data are shown on scales comparable to that in previous studies [36,39,40].

When extracting the critical shear stress and critical shear strain of the side stream blends from the end of the LVR, all samples were condensed in the bottom left corner of the texture map, which is ascribed to ‘mushy’ texture properties (Figure 7A). Using the cross-over of G’ and G’’ to create the texture map shifted the data points to considerably higher stress and strain values (Figure 7B), as expected from the high flow transition index, indicating a much higher strain at the cross-over than at the end of the LVR (see Figure 5B). However, when compared to absolute values in previous studies [36,39,40], the samples can still be considered ‘mushy’. The insets in Figure 7 show magnifications of the bottom left corners of the texture maps to allow a better distinction of the different samples. In view of the rather large error bars, the small differences might be considered negligible. There seems to be a slight tendency towards a mushier texture with fermentation (squares vs. circles). However, as process-induced changes in the texture attributes occur usually over several decades in critical shear stress and/or critical shear strain [37,38], it can be concluded that fermentation had no effect on the large deformation properties of the side stream blends.

### 3.4. Visual Appearance and Microstructure

**Figure 8:**
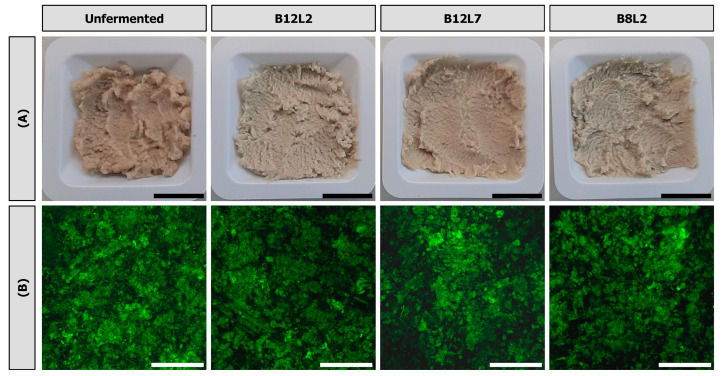
Representative (**A**) photographs and (**B**) confocal microscopy images of heat-treated (121 °C, 5 min) 225 g/kg sunflower seed press cake in reconstituted whey (60 g/kg) before and after fermentation with different co-cultures of lactic acid bacteria (*Lactococcus lactis* strain B12; *Lacticaseibacillus paracasei* strain B8) and yeasts (*Kluyveromyces lactis* strain L2; *Kluyveromyces marxianus* strain L7) at 26 °C for 48 h. Length of black and white bars corresponds to 1.5 cm and 200 µm, respectively.

Figure 8A shows representative images of the side stream blends before and after fermentation. All samples were semi-solid and spreadable, agreeing with the ‘mushy’ texture that was identified in the texture map (see Figure 7). Slight differences in the colour of the side stream blends were noticeable, as was also confirmed by the colour measurements (Table 1). The colour parameters of the unfermented blend are in good agreement with results from a previous study on side stream blends with similar compositions, but which were heated to 120 °C using a different procedure [8]. The lightness (L*) increased significantly with fermentation, although this was only statistically significant for the side stream blends fermented with B12L2. All fermented samples had significantly lower a* values of around 0, indicating a stronger red colour of the unfermented blend, which was changed slightly towards green with fermentation. A statistically significant decrease in b* was only observed for fermentation with B12L2. However, all samples showed b* > 0, indicating a pronounced yellow colour, as also visible from the photographs (Figure 8A). It can be concluded that fermentation with all co-cultures resulted in a lighter and less red colour, where the fermentation with B12L2 showed the strongest impact. This colour change may be related to oxidative reactions, in particular the non-enzymatic oxidation of the chlorogenic acid, the main phenolic compound of sunflower seed press cake, which generates a green colour of the substrate. However, this reaction is favoured at neutral pH rather than in acidic conditions [41]. A green colour of the sunflower seed press cake could also be associated with the combination of chlorogenic acid in the press cake and whey components [42]. On the other hand, the acidification could be responsible for the turn towards the yellow colour.

The microstructure of the unfermented and fermented side stream blends is shown in Figure 8B. All samples exhibited a homogeneous microstructure with some visible interstitial areas, which occurred most likely during sampling after autoclaving. There was no visible effect of fermentation on the microstructure of the side stream blends. In a previous study [8], the heat treatment of side stream blends under quiescent conditions resulted in the formation of a homogeneous network, whereas heating under continuous stirring caused the formation of protein aggregates of 10–100 µm dispersed in an aqueous matrix. In the present study, the side stream blends were heated in an autoclave without agitation but mixed using a kitchen blender after the heat treatment in order to inoculate them with the co-cultures. This mixing step, while not causing a breakdown of the network structure, would confirm that the side stream blends are composed of a dense, hydrated matrix of arrested particles, which behaves like a particle gel at low strain amplitudes (see Figure 5A), but which flows at larger strains (Figure 5B) and recovers when arrested again.

### 3.5. Storage Stability of the Fermented Blends

Parts of the fermented blends were stored at 4 °C and analysed again after 7 d of storage for their viable cell counts, pH, and rheological properties. While the pH (Table 2) and the cell counts of lactic acid bacteria and yeasts did not significantly change during storage, bacterial contaminants were detected in some of the stored samples (Table 2). In particular, all side stream blends fermented with B8L2 contained substantial levels of bacterial contaminants, which was not surprising, as this co-culture resulted in a significantly delayed acidification (Figure 1C), and mesophilic aerobic bacterial contaminants were already found in the freshly prepared blends after fermentation for 48 h (Section 3.1.3).

G’ and tan δ were barely changed during storage; statistically significant differences between the fresh and stored blends were only observed for two of the samples fermented with B8L2 (Table 2). However, the values were still within the overall range of G’ and tan δ that was obtained for all samples (see Figure 6A), indicating that the effect of storage was minimal.

## 4. Conclusions

### 4.1. Fermentation of Side Stream Blends

Co-cultures of lactic acid bacteria and yeasts can be particularly beneficial in fermenting food substrates, as acidification and ethanol production are combined to create micro-environmental conditions hostile to the proliferation of pathogens, spoilage bacteria, and moulds. In this study, three selected microbial consortia composed of one lactic acid bacteria strain (*Lactococcus lactis* strain B12 or *Lacticaseibacillus paracasei* strain B8) and one yeast strain (*Kluyveromyces lactis* strain L2 or *Kluyverumyces marxianus* strain L7) were tested to ferment blends of sunflower seed press cake and cheese whey. The fermentative process was assessed with regard to cell growth, acidification, the metabolisation of sugars, and the rheology and structure of the fermented blends. The growth of *Lacticaseibacillus paracasei* strain B8 was delayed compared to that of *Lactococcus lactis* strain B12, resulting in a slower acidification of the blend and the growth of bacterial contaminants. In this regard, the availability of nutrients present in the side stream blends was redundant with respect to the needs of the inoculated microorganisms, causing a competition phenomenon between *Lacticaseibacillus paracasei* strain B8 and *Kluyveromyces lactis* strain L2 rather than a synergistic relationship, as observed in some traditional fermented foods. Fermentation with neither of the co-cultures affected the microstructure and rheological properties of the blends, which could be because the heat treatment at 121 °C prior to inoculation already formed a heat-set composite gel of proteins and polysaccharides. This finding is remarkable, as it allows one to predict the properties and processability of the fermented blends already based on the unfermented material. From the experiments, it was concluded that the co-culture composed of *Lactococcus lactis* strain B12 and *Kluyveromyces lactis* strain L2 was the most suitable for fermenting the blends of sunflower seed press cake and cheese whey, as (1) it was capable of acidifying the blend to pH < 4.8 within 24 h, (2) the fermented blend remained free of bacterial contaminants and moulds during fermentation as well as after 7 d of storage at 4 °C, and (3) the viable cell counts of lactic acid bacteria and yeasts did not decrease during 7 d of storage at 4 °C. Therefore, this microbial association was selected for the performance of further studies on the post-processing of fermented blends to further foster the development of safe and suitable food applications.

### 4.2. Further Considerations and Outlook

Blending and fermenting side streams holds great potential for developing sustainable and palatable foods or food ingredients and reducing food waste. The protocol proposed in this work also allows fresh water to be saved through using an aqueous, food-grade side stream. In fact, this approach might also be suitable for valorising other side streams, such as rapeseed and flaxseed press cake, brewer’s spent grain, and okara as solid components, as well as butter milk and milk ultrafiltration permeate as liquid components. Nevertheless, a precise evaluation of the composition obtained by blending side streams must be carried out in order to design blends that can provide a healthy balance of nutrients as well as acceptable sensory properties. Press cakes from other oilseed varieties or from seeds that contain a higher percentage of hulls might show a considerably different composition, especially in terms of the protein/fibre ratio as well as anti-nutritional components and off-flavours. Furthermore, an assessment of the digestibility of the protein fractions should be carried out to investigate the nutritional quality of the designed product.

Since the selected microorganisms are mesophilic, fermentation at room temperature is possible, which may eliminate the need for energy-intensive incubators. The potential tuning of the matrices in terms of structure, texture, and acidification through the exploitation of the enzymatic activities of different microorganisms can pave the way for editing the fermentation process and lead to the success of the new products. Detailed cost and life cycle analyses will be carried out towards the end of the whole project to underline the relevance of our research (https://ferblend.webspace.tu-dresden.de/; accessed on 28 October 2023) [3].

However, smaller factories might not be prepared for utilising side streams such as oilseed press cakes and cheese whey in other ways than they currently do. Overcoming this issue might require incentives from public or private sectors, which encourage manufacturers to invest in new equipment and infrastructure that facilitate the valorisation of their side streams. Finally, it might be necessary consider regulatory issues such as approvals as novel foods.

In following studies of the project, sensory analyses and consumer acceptance will be carried out to demonstrate the market potential of the developed products [3]. The way of marketing products obtained from food processing side streams needs to be carefully evaluated to avoid confusion and thus rejection by the consumers directly at the beginning. This issue, however, should be addressed in a broader framework than the current research project, and is in fact also being investigated by researchers from economical sciences [43].

## Figures and Tables

**Table 1:** Colour parameters of heat-treated (121 °C, 5 min) 225 g/kg sunflower seed press cake in reconstituted whey (60 g/kg) before and after fermentation with different co-cultures of lactic acid bacteria (*Lactococcus lactis* strain B12; *Lacticaseibacillus paracasei* strain B8) and yeasts (*Kluyveromyces lactis* strain L2; *Kluyveromyces marxianus* strain L7) at 26 °C for 48 h. Different letters within the same row indicate statistically significant differences between the samples (*p* < 0.05).

	Unfermented	B12L2	B12L7	B8L2
L* (−)	66.5 ± 3.3 ^a^	75.0 ± 0.2 ^b^	72.3 ± 1.8 ^a,b^	72.8 ± 1.2 ^a,b^
a* (−)	3.4 ± 0.1 ^a^	0.0 ± 0.4 ^b^	0.6 ± 1.0 ^b^	0.6 ± 0.7 ^b^
b* (−)	16.7 ± 0.4 ^a^	13.7 ± 0.2 ^b^	14.7 ± 1.2 ^a,b^	14.6 ± 2.2 ^a,b^

**Table 2:** pH, cell count of bacterial contaminants, storage modulus (G’), and loss factor (tan δ) of heat-treated (121 °C, 5 min) 225 g/kg sunflower seed press cake in reconstituted sweet whey (60 g/kg) fermented with different co-cultures of lactic acid bacteria (*Lactococcus lactis* strain B12; *Lacticaseibacillus paracasei* strain B8) and yeasts (*Kluyveromyces lactis* strain L2; *Kluyveromyces marxianus* strain L7) at 26 °C after storage at 4 °C for 7 d. * indicates statistically significant differences between the fresh and stored samples (*p* < 0.05).

Co-Culture	Fermentation Time (h)	pH (−)	Bacterial Contaminants (Log_10_ CFU/g)	G’ (Pa)	tan δ (−)
B12L2	12 24 48	5.23 ± 0.62 4.78 ± 0.35 4.57 ± 0.25	<2 <2 <2	18,490 ± 5890 21,755 ± 7115 23,300 ± 1580	0.22 ± 0.01 0.22 ± 0.01 0.22 ± 0.01
B12L7	12 24 48	5.08 ± 0.21 4.88 ± 0.18 4.65 ± 0.08	<2 2.48 ± 0.34 * <2	19,105 ± 6715 14,540 ± 3800 17,370 ± 4520	0.22 ± 0.01 0.22 ± 0.01 0.22 ± 0.01
B8L2	12 24 48	5.96 ± 0.04 5.50 ± 0.31 4.67 ± 0.21	2.96 ± 1.36 * 4.67 ± 0.28 * 4.80 ± 0.28 *	18,620 ± 1340 * 27,425 ± 2775 * 24,925 ± 3525	0.20 ± 0.00 0.21 ± 0.00 * 0.21 ± 0.00

## Data Availability

The data presented in this study are available on request from the corresponding author.
